# Electronic Media Device Usage and Its Associations With BMI and Obesity in a Rapidly Developing City in South China

**DOI:** 10.3389/fpubh.2020.551613

**Published:** 2021-01-08

**Authors:** Ying Qiu, Yao Jie Xie, Liping Chen, Shao Ling Wang, Hualu Yang, Zhenzhen Huang, Ping Liu, Beirong Mo

**Affiliations:** ^1^Department of Nursing, Huazhong University of Science and Technology Union Shenzhen Hospital, Shenzhen, China; ^2^School of Nursing, The Hong Kong Polytechnic University, Hong Kong, China

**Keywords:** electronic device, screen-based, BMI, Chinese, obesity

## Abstract

**Objective:** This study aimed to evaluate the television (TV) viewing and computer and mobile phone usage duration in a community sample of Chinese adults and examine their associations with BMI and obesity.

**Methods:** We conducted a community-based health needs assessment study from February to December 2018 among 2,873 Chinese adults in Nanshan District of Shenzhen, China. We used self-administered questionnaires to collect the data from 24 community health service centers in Nanshan District. The participants individually recorded the time they spent watching TV and using computers and mobile phones. They also answered questions about their sociodemographic and lifestyle factors. We measured their height and weight by using ultrasonic height and weight scales. Then, we calculated and categorized their BMI in accordance with the standards prescribed by the World Health Organization for Asians.

**Results:** Most of the participants were young adults (aged 18–44 years, 74.2%). The overall prevalence of obesity was 15.30%. The prevalence of TV, computer, and mobile phone usage was 75.5, 71.2, and 93.8% in females and 71.1, 75.7, and 94.2% in males, respectively. The youth (18–24 years) and the elderly (65 years or older) had the longest time using mobile phones (3.78 ± 2.51 h) and watching TV programs (2.12 ± 1.38 h), respectively. Longer usage of computers and mobile phones was evident in males (*p* < 0.05) and participants with a high education level (*p* < 0.01). The univariate analysis results showed an association between high BMI and obesity and short duration of using computers and mobile phones (all *p* < 0.05). By contrast, no significant associations were found between the length of TV program viewing and mobile phone usage and BMI (*p* > 0.05). After we adjusted for potential confounders, we found that computer usage time and the overall usage time of the three electronic devices had an inverse association with BMI (*p* < 0.05).

**Conclusions:** Mobile phones were the most popular electronic device in Nanshan residents of Shenzhen. Unlike most previous studies, we found an inverse association between screen time and BMI. Nevertheless, scholars should conduct further studies to explore this association. Overall, we strongly encourage the appropriate use of electronic devices.

## Introduction

Nowadays, mass media, especially those Internet-based communication tools, are a significant infrastructure of the modern information society. The usage of screen-based electronic devices, such as watching television (TV) programs and using computers and mobile phones, has become an integral part of people's lifestyle. Approximately 90% of the population in the United States, the United Kingdom, and China watch TV programs, and 80% use smartphones (1). In the past 10 years, the proportion of the population using mobile Internet devices increased from 45.9 to 99.1% in China (2). The increasing use of electronic media devices highlights the importance of investigating the potential effect of their usage on human health.

Obesity is a massive threat to current human health. The World Health Organization (WHO) reported that overweight and obesity in adults (≥18 years) had shown a continuous upward global trend over the past four decades, with ~2 billion adults suffering from overweight and more than half a billion with obesity in 2016 (3). In China, the prevalence of overweight and obesity (≥18 years) increased from 29.9 to 42% between 2002 and 2012 (4). Thus, preventing the increase in cases of obesity has become one of the most significant public health issues, highlighting the need to identify more contributors to such a condition in modern society.

The most common contributors to obesity include increased availability of processed foods, low physical activity (PA) and sedentary lifestyle (5). Scholars identified a significant association between the extended time in watching TV programs and using computers, which are the typical sedentary lifestyle, and elevated risk of obesity in adults (6–10) and adolescents (11–14). A study conducted in Xian, China, also showed that Internet addiction by using smartphones and computers is an independent risk factor of obesity among middle-school students (15). Using computers to access the Internet for leisure also strongly contributes to the variation of overweight and obesity among adults. However, such a contribution is mainly independent of doing PA for leisure (16).

Although previous studies focused on the associations between screen-based electronic device usage and obesity, most of them investigated only one or two electronic devices. Thus, the electronic device usage that contributes most to the changes in BMI is still unknown, and the association between the overall device usage and BMI/obesity remains underexplored.

The simultaneous use of multiple electronic screen devices has become an increasing trend in modern society. Hence, a comprehensive investigation of the usage of several screen-based electronic media devices may help us to understand further the overall situation. Moreover, the knowledge of the associations of obesity with multiple electronic screen devices remains little among the Chinese population. Accordingly, we conduct this study to investigate the status of TV program viewing and computer and mobile phone usage in a large community sample of Chinese adults in Shenzhen, which is a rapidly developing city in South China. We also examine the associations of BMI and obesity with the above population's usage duration of these three electronic devices.

## Methods

### Study Design and Setting

The data analyzed in this study were from a community-based survey conducted in 24 health service centers in Nanshan District of Shenzhen from February to December 2018. This survey was a health needs assessment study for the urban and representative adult population in the district. It assesses the lifestyle behaviors (dietary habits, sleep, PA, electronic device usage, smoking, and drinking) and health needs in Nanshan residents, understands the relevant health information-seeking behaviors, and identifies the barriers for a healthy lifestyle in vulnerable groups. The Huazhong University of Science and Technology (HUST) Union Shenzhen Hospital manages the 24 selected community health service centers. They aim to provide primary health care to the community residents, solve typical health problems in the community, and meet the necessary health demands of the community. They offer various health services, including vaccination, rehabilitation, health education, birth control, post-partum follow-up, and chronic disease management. The service population is mainly composed of women, children, elderly, patients with chronic diseases, persons with disabilities, and some vulnerable groups. The 24 selected health service centers serve 378,799 community members. These people account for 36% of the Nanshan District residents. On average, these 24 health service centers served 2,871 people per day in 2018. The HUST Union Shenzhen Hospital and the Human Research Committee of Hong Kong Polytechnic University (PolyU) approved this study (HSEARS20180521004). All participants also signed the informed consents.

### Study Population

We regarded the Nanshan District residents who visited one of the 24 selected community health service centers as the potential participants of our study. The inclusion criteria were (1) 18 years or older, (2) able to read and write Chinese or speak Cantonese or Putonghua, and (3) currently residing in Nanshan District. The exclusion criteria were (1) living in Nanshan District for less than a year, (2) psychologically or physically unable to communicate, and (3) unable to provide informed consents.

#### Sample Size, Sampling, and Recruitment Procedure

On the basis of the Nanshan population size (18 years or older) of 1,356,307 at the end of 2016 (17), we assumed that the expected prevalence of all chronic diseases was 30%, with consideration of 5% type I error. Thus, we needed 2,800 participants to achieve 80% power for our study. We used a stratified random sampling approach to recruit participants. First, we determined the number of needed participants in each age group (aged 18–24, 25–34, 35–44, 45–54, 55–64, and 65 years or older) by referring to the distribution of age and gender in Nanshan population (17). HUST Union Shenzhen Hospital had records of the Nanshan residents who had registered at the community health service centers. These residents visited the service centers for body check or health consultation regularly. In March 2018, the principal investigator from PolyU conducted a workshop for all head nurses working in the 24 selected community centers to train them about the recruitment process and data collection approaches. Then, the head nurses trained their centers' nurses for subject recruitment and data collection. Since May 2018, the trained nurses started to randomly invite potential participants who visited the service centers to join the study. Subsequently, they screened these potential participants following the inclusion and exclusion criteria. We considered those who met the eligibility criteria, and the eligible participants in our study provided written informed consents. Afterward, we conducted the data collection process. The recruitment lasted for 6 months.

### Measures

#### Measurement for the Duration of Watching TV Programs and Using Computers and Mobile Phones

In the questionnaire, we asked the participants about their electronic media device usage: “In daily time, on average, how many hours do you spend on watching TV programs and using computers and mobile phones?” The participants indicated the hours (to the nearest 0.1 h) they spent on these three devices. We considered 0 as no use. Finally, we summarized the total hours spent on the three electronic devices together.

#### Assessment of BMI and Obesity

Ultrasonic height and weight scales are available in the lobbies of the community health service clinics. We asked the participants to measure their weight and height twice independently. Then, they calculated and recorded their average weight and height in their questionnaire to the nearest 0.1 kg and 0.1 m, respectively. We calculated BMI as body weight in kilograms divided by squared body height in meters (BMI = kg/m^2^). According to the standards prescribed by WHO for Asians (18), we defined obesity, overweight, normal weight, and underweight as BMI ≥ 25 kg/m^2^, 23 kg/m^2^ ≤ BMI < 25 kg/m^2^, 18.5 kg/m^2^ ≤ BMI < 23 kg/m^2^, and BMI < 18.5 kg/m^2^, respectively. Then, we grouped the last three categories of BMI as non-obese (BMI < 25 kg/m^2^) as a contrast group for obesity in our analysis.

#### Measurement of Sociodemographic and Lifestyle Factors

We collected via the questionnaire the sociodemographic data of the participants. Specifically, we obtained the following categories: age groups (18–24, 25–34, 35–44, 45–54, 55–64, 65 years or older), gender (male and female), work status (employed, unemployed, students, and retired), marital status (married, cohabiting, divorced, separated, widowed, and unmarried), family monthly income (<4,000, 4,000–9,999, 10,000–19,999, 20,000–29,999, 30,000–39,999, 40,000–59,999, and ≥60,000 CNY), and education level (primary school and below, secondary school, college, master's degree and above).

The lifestyle factors measured in our study included PA, frequency of breakfast, smoking status, and drinking habits. We measured PA by using the items adopted from the International Physical Activity Questionnaire. We measured the frequency and duration of moderate and vigorous PA separately. Then, we calculated the total weekly time spent in moderate-to-vigorous PA as the sum of these two activities. Following the PA guidelines prescribed by WHO (19), we defined enough PA as total PA ≥150 min/week. We regarded those <150 min/week as not enough PA for analysis. We also measured the frequency of breakfast in this study by using five categories (never, 1–2 times/week, 3–4 times/week, 5–6 times/week, and every day). Finally, we assessed the smoking status and drinking habits as current smoker/drinker and non-smoker/drinker.

### Data Collection Procedure

The trained nurses in each community center collected the data from the participants. The head nurses prepared the questionnaires in advance. They used a worksheet to record data collection progress and submitted their records to the hospital nursing department monthly. The project investigators from PolyU and HUST Union Shenzhen Hospital closely monitored the progress of data collection. After providing written informed consent, the eligible participants received the self-administered questionnaire from the community nurses. The nurses also conducted a face-to-face interview with those who could not read (e.g., the elderly). A valid questionnaire had no more than 10% of missing data. Thus, we regarded those with more than 10% missing data as partial or incomplete questionnaires.

### Data Analysis

We adopted mean and standard deviation (SD) for the continuous variables and count and percentage for the categorical variables to describe the basic characteristics of the participants.

To examine the device usage and obesity status of the participants, we presented their duration of usage (hours), BMI, and obesity status (yes/no) through mean ± SD or frequency (*n* and %) where appropriate. Then, we analyzed their device usage and obesity status in accordance with different demographical groups by using one-way analysis of variance (ANOVA) or independent *t*-test for the continuous variables and χ^2^-test for the categorical variables.

To test the association between BMI/obesity and the usage of the three devices, we implemented a univariate analysis. We compared the overall time of using the devices and the time for each device between four BMI groups by ANOVA and between obese and non-obese participants by independent *t*-test. We also performed multiple linear regression models to determine whether the three devices usage had independent associations with BMI and identify the strength and direction of the associations. Finally, we adjusted the demographical and lifestyle variables that had significant associations with BMI/obesity or device usage time in previous analysis in the regression models. We used SPSS version 25.0 (SPSS Institute) in all data analyses and considered a significance level of 0.05.

## Results

### Basic Characteristics of the Participants

We disseminated 3,000 questionnaires to the eligible participants and received 2,873 valid questionnaires in return. The response rate was 95.8%. Table 1 shows the basic characteristics of the participants. Most of the participants in our study were young adults (18–44 years old), accounting for 74.16% of the total population. The proportion of young females was slightly higher than the young males (75.5 vs. 73.0%, *p* < 0.05). Most of the participants were married (74.48%) and employed (86.81%), and approximately two-thirds had university or higher education. Women had a lower education level than men (*p* < 0.05). The marital status (*p* > 0.05) and family monthly income (*p* > 0.05) of both genders did not differ significantly. Two income levels, namely, 10,000–19,999 CNY (30.63%) and 40,000–49,999 CNY (27.65%), were proportionally higher than others. Half of the participants did not have enough PA, and 59.8% reported to eat breakfast every day. Women were less physically active but ate breakfast more frequently than men (all *p* < 0.01). Moreover, 11.0% of the participants were current smokers, and 25.9% consumed alcohol. Men were more likely to consume cigarettes and alcohol than women (*p* < 0.01).

**Table 1:** Basic characteristics of the participants.

	***n***	**Gender**		***P-*value**
		**Female**	**Male**	
**Age**	2,864			0.034
18–24 years		172 (12.6%)	172 (11.5%)	
25–34 years		480 (35.2%)	519 (34.6%)	
35–44 years		377 (27.7%)	404 (26.9%)	
45–54 years		196 (14.4%)	260 (17.3%)	
55–64 years		104 (7.6%)	90 (6.0%)	
65 years and above		33 (2.4%)	57 (3.8%)	
**Work status**	2,843			<0.001
Employed		1,129 (83.6%)	1,339 (89.7%)	
Student		27 (2.0%)	37 (2.5%)	
Retired		88 (6.5%)	90 (6.0%)	
Unemployed		106 (7.9%)	27 (1.8%)	
**Marital status**	2,829			0.462
Single		335 (24.9%)	387 (26.1%)	
Married		1,011 (75.1%)	1,096 (73.9%)	
**Education level**	2,834			0.011
Primary school and below		43 (3.2%)	28 (1.9%)	
Secondary school		392 (29.0%)	423 (28.5%)	
College		873 (64.6%)	956 (64.5%)	
Master's degree and above		43 (3.2%)	76 (5.1%)	
**Family monthly income (CNY)**	2,814			0.196
<4,000		104 (7.8%)	99 (6.7%)	
4,000–9,999		93 (6.9%)	124 (8.4%)	
10,000–19,999		417 (31.1%)	445 (30.2%)	
20,000–29,999		172 (12.8%)	217 (14.7%)	
30,000–39,999		61 (4.5%)	84 (5.7%)	
40,000–59,999		378 (28.2%)	400 (27.2%)	
≥60,000		45 (3.4%)	45 (3.1%)	
Do not know		71 (5.3%)	59 (4.0%)	
**PA level**^a^	2,517			<0.001
Not enough		633 (54.2%)	584 (43.3%)	
Enough		534 (45.8%)	766 (56.7%)	
**Frequency of breakfast in last week**	2,796			<0.001
Never		16 (1.2%)	41 (2.8%)	
1–2 times		86 (6.5%)	124 (8.5%)	
3–4 times		135 (10.1%)	177 (12.1%)	
5–6 times		233 (17.5%)	311 (21.2%)	
Everyday		861 (64.7%)	812 (55.4%)	
**Smoking**	2,722			<0.001
Yes		26 (2.0%)	272 (18.8%)	
No		1,252 (98.0%)	1,172 (81.2%)	
**Drinking**	2,747			<0.001
Yes		170 (13.1%)	542 (37.3%)	
No		1,124 (86.9%)	911 (62.7%)	

a *The total time spent in MVPA was calculated as the sum of these two activities following the PA guidelines for health benefits. Insufficient and sufficient MVPA were then defined as <150 min/week and ≥150 min/week, respectively, as prescribed by WHO*.

### Daily Duration of TV Program Viewing and Computer and Mobile Phone Usage Among the Participants

**Figure 1:**
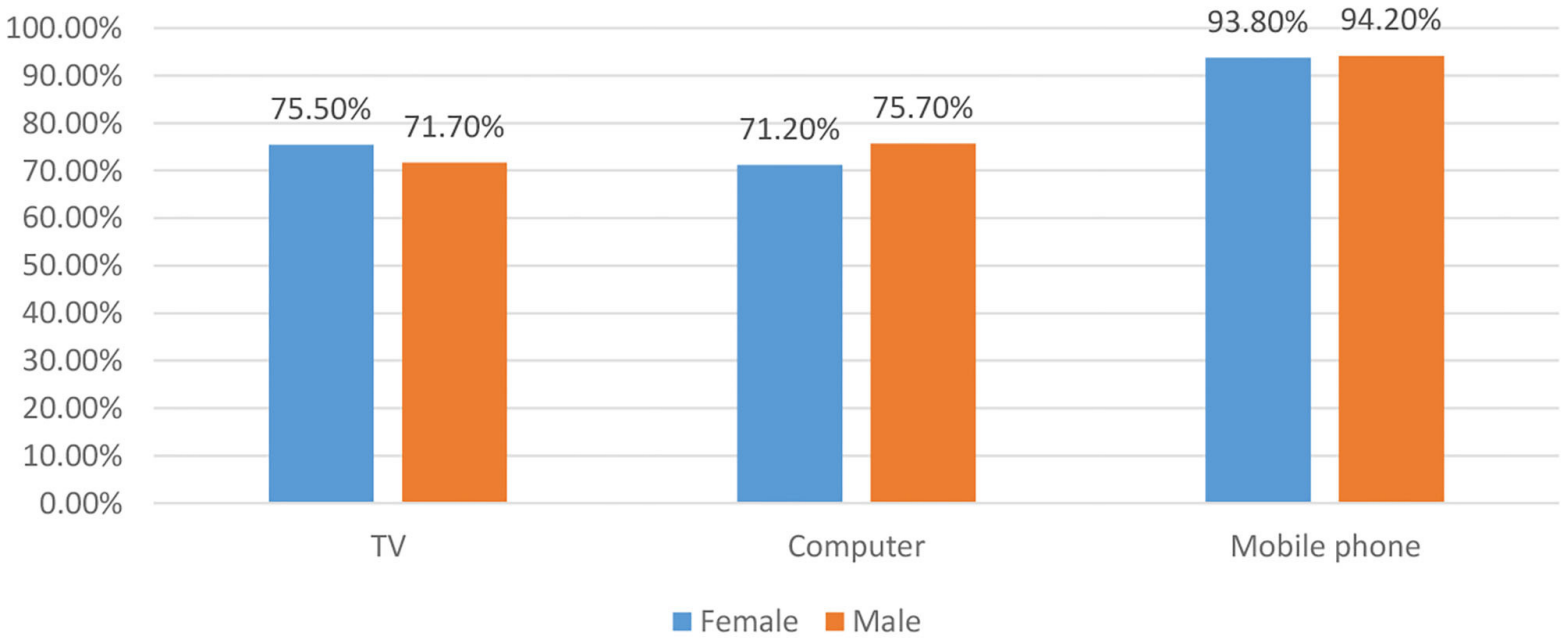
The proportion of participants using one of the three devices according to gender.

Table 2 shows the average daily hours of TV program viewing and computer and mobile phone usage and the overall hours of using three electronic devices of the participants. Only 3.13% of the participants did not use any one of the three devices. Figure 1 shows the proportion of the participants using one of the three devices by gender. More than 90% of the participants used mobile phone daily. Although the male participants were not different from their female counterparts in terms of mobile phone usage (94.2 vs. 93.8%, *p* > 0.05), they comprised a larger proportion of computer users (75.7 vs. 71.2%, *p* < 0.05). In line with the above results, the female participants watched TV programs longer than the male participants (71.7 vs. 75.5%, *p* < 0.01).

**Table 2:** Associations between BMI and obesity and the time spent by the participants using the three electronic media devices.

	**TV program viewing time (daily hours), mean (SD)**	***P***	**Computer usage time (daily hours), mean (SD)**	***P***	**Mobile phone usage time (daily hours), mean (SD)**	***P***	**Overall usage time of the three devices (daily hours), mean (SD)**	***P***	**BMI (kg/m^**2**^), mean (SD)**	***P***	**Obesity, n (%)**	***P***
**Age**		<0.001		<0.001		<0.001		<0.001		<0.001		<0.001
18–24 years	1.02 (1.17)		2.90 (2.77)		3.78 (2.51)		7.29 (3.88)		21.2 (3.35)		25 (7.3%)	
25–34 years	1.10 (1.06)		2.96 (2.85)		3.33 (2.59)		6.97 (4.10)		22.0 (3.17)		10.6 (10.7%)	
35–44 years	1.01 (1.15)		2.05 (2.41)		2.67 (2.17)		5.6 (3.80)		22.8 (3.08)		13.0 (16.8%)	
45–54 years	1.15 (1.03)		1.65 (2.39)		2.50 (2.23)		4.86 (3.35)		23.5 (3.32)		110 (24.4%)	
55–64 years	1.80 (1.87)		1.26 (2.16)		1.90 (1.76)		4.64 (3.39)		23.4 (2.64)		50 (26.2%)	
65 years and above	2.12 (1.38)		1.18 (2.02)		2.03 (2.61)		4.94 (4.01)		23.2 (2.36)		14 (15.6%)	
**Gender**		0.181		0.001		0.002		0.011		<0.001		<0.001
Female	1.19 (1.18)		2.15 (2.60)		2.78 (2.27)		5.88 (3.96)		21.9 (3.20)		159 (11.8%)	
Male	1.12 (1.22)		2.50 (2.71)		3.07 (2.56)		6.26 (3.93)		23.1 (3.10)		275 (18.5%)	
**Work status**^**a**^		<0.001		<0.001		<0.001		<0.001		0.031		0.169
Employed	1.06 (1.07)		2.50 (2.71)		3.05 (2.42)		6.26 (3.98)		22.50 (3.17)		364 (14.9%)	
Unemployed	1.67 (1.41)		1.32 (2.12)		2.18 (2.36)		4.86 (3.50)		22.85 (3.50)		66 (17.6%)	
**Marital status**^**b**^		<0.001		<0.001		<0.001		<0.001		<0.001		<0.001
Single	0.99 (1.08)		3.12 (2.87)		3.74 (2.73)		7.36 (4.03)		21.7 (3.29)		55 (7.6%)	
Married	1.21 (1.24)		2.07 (2.52)		2.65 (2.21)		5.67 (3.82)		22.8 (3.15)		375 (18.0%)	
**Family monthly**		0.011		<0.001		0.044		<0.001		0.043		0.002
<4,000	1.42 (1.21)		2.11 (2.84)		3.04 (3.07)		5.83 (4.22)		22.9 (3.55)		42 (20.5%)	
4,000–9,999	0.97 (1.02)		2,54 (2.63)		3.10 (2.34)		6.53 (4.43)		22.0 (2.67)		26 (12.0%)	
10,000–19,999	1.16 (1.17)		2.49 (2.67)		3.08 (2.44)		6.46 (3.94)		22.4 (3.25)		111 (12.9%)	
20,000–29,999	1.15 (1.09)		2.61 (2.85)		2.82 (2.19)		6.20 (3.93)		22.8 (2.96)		75 (19.4%)	
30,000–39,999	1.09 (0.90)		2.87 (2.86)		2.77 (2.16)		6.33 (3.96)		22.7 (2.94)		28 (19.3%)	
40,000–59,999	1.19 (1.39)		1.99 (2.43)		2.80 (2.15)		5.73 (3.67)		22.5 (3.23)		112 (14.6%)	
≥60,000	1.01 (1.02)		1.69 (2.35)		2.45 (2.50)		4.73 (3.44)		22.8 (3.09)		20 (22.2%)	
Do not know	1.03 (1.16)		2.58 (2.96)		3.29 (3.21)		6.03 (4.22)		22.1 (4.11)		13 (10.2%)	
**Education level**		<0.001		<0.001		<0.001		<0.001		<0.001		<0.001
Primary school and below	1.93 (1.54)		1.00 (2.06)		1.66 (2.15)		4.16 (3.69)		24.0 (4.62)		25 (34.2%)	
Secondary school	1.34 (1.40)		1.34 (1.99)		2.56 (2.40)		5.00 (3.63)		23.0 (3.48)		157 (19.4%)	
College	1.08 (1.09)		2.76 (2.78)		3.14 (2.36)		6.61 (3.96)		22.3 (3.02)		236 (13.0%)	
Master's degree and above	0.77 (0.80)		3.37 (3.09)		3.03 (2.70)		6.62 (4.05)		22.1 (2.77)		15 (12.7%)	
**Smoking**		0.06		0.243		0.183		0.941		<0.001		0.007
Yes	1.05 (1.02)		2.14 (2.50)		3.14 (2.36)		6.10 (3.76)		23.2 (2.89)		61 (20.5%)	
No	1.17 (1.22)		2.34 (2.66)		2.94 (2.44)		6.09 (3.94)		22.4 (3.14)		350 (14.6%)	
**Drinking**		0.049		0.004		0.757		0.033		<0.001		<0.001
Yes	1.07 (1.13)		2.60 (2.69)		2.93 (2.24)		6.37 (3.82)		22.9 (3.05)		141 (19.9%)	
No	1.17 (1.22)		2.25 (2.64)		2.97 (2.50)		6.00 (3.97)		22.3 (3.11)		266 (13.2%)	
**PA level**^**c**^		0.118		<0.001		0.797		0.001		0.976		0.35
Not enough	1.09 (1.15)		2.13 (2.58)		2.96 (2.49)		5.82 (3.95)		22.5 (3.16)		189 (15.6%)	
Enough	1.17 (1.22)		2.53 (2.65)		2.94 (2.36)		6.35 (3.93)		22.5 (3.02)		184 (14.3%)	
**Frequency of breakfast in last week**		0.255		0.041		<0.001		<0.001		<0.001		0.012
Never	0.87 (1.33)		1.86 (2.53)		2.88 (2.58)		5.03 (4.03)		23.8 (5.61)		15 (26.8%)	
1–2 times	1.21 (1.35)		2.43 (2.69)		3.18 (3.00)		6.15 (4.00)		22.4 (3.01)		25 (12.0%)	
3–4 times	1.07 (0.98)		2.40 (2.56)		3.41 (2.73)		6.51 (3.95)		22.4 (2.57)		41 (13.2%)	
5–6 times	1.17 (1.06)		2.63 (2.72)		3.22 (2.48)		6.62 (4.01)		22.1 (2.64)		70 (12.9%)	
Everyday	1.07 (1.27)		2.25 (2.67)		2.72 (2.23)		5.86 (3.90)		22.6 (3.35)		275 (16.5%)	

a *Unemployed, students, and retired participants were all defined as unemployed*.

b *Couple and cohabiting participants were defined as married, and divorced, separated, widowed, and unmarried participants were all defined as single*.

c *The total time spent in moderate-to-vigorous physical activity (MVPA) was calculated as the sum of these two activities following physical activity guidelines for health benefits. Insufficient and sufficient MVPAs were then defined as <150 and ≥150 min/week, respectively, as prescribed by WHO*.

The TV program viewing time significantly increases with age, and thus, the elderly had reported the longest time watching TV programs (*p* < 0.001). By contrast, the computer and mobile phone usage time decreases with age (both *p* < 0.01). The youth (18–24 years old) had reported the longest time using mobile phones (3.78 ± 2.51 h) and the overall three devices (7.29 ± 3.88 h). The male participants had longer usage of computers and mobile phones than their female counterpart (*p* < 0.05). Those employed and are single spent less time watching TV programs but more time using computers and mobile phones (all *p* < 0.01). We also observed significant differences among different levels of family monthly income for the usage duration of the three devices (all *p* < 0.05). Regarding the association between education level and device usage, those with high education level spent less time watching TV programs but more time using computers and mobile phones (all *p* < 0.01). We found no significant difference in terms of smoking status (all *p* > 0.05). Current drinkers spent less time watching TV programs and more time using computers (both *p* < 0.05). Those who had enough PA used computers longer in their daily lives than those without enough PA (*p* < 0.01). Moreover, those who do not eat breakfast spent less time using computers and mobile phones than those who eat it every day (both *p* < 0.05). For the overall time of using the three devices, we observed significant differences in terms of the demographic and lifestyle variables (all *p* < 0.05), except for smoking status (*p* > 0.05).

### BMI and Obesity Status Among the Participants and Their Associations With the Usage Duration of the Three Electronic Devices

The mean BMI was 21.9 (±3.20) kg/m^2^ in females and 23.1 (±3.10) kg/m^2^ in males (*p* < 0.01). The overall prevalence of obesity was 15.30% among the participants, with higher cases in males than in females (18.5 vs. 11.8%, *p* < 0.01). Table 2 shows the BMI and obesity status by the demographical variables. We found significant differences among the participants in terms of age, gender, work status, marital status, family monthly income, education level, smoking status, drinking habits, and frequency of breakfast (all *p* < 0.05). The male participants had a higher prevalence of obesity than their female counterparts (11.8 vs. 18.5%). Those unemployed, married, smokers, and drinkers had reported high BMI and mostly suffered from obesity. The BMI and obesity prevalence decrease with the increase in education and PA levels. The participants who never ate breakfast and those who eat it every day were likely to be fat (*p* < 0.05).

Table 3 shows the associations between BMI and obesity and the usage duration of the three devices. High BMI had an association with short time using computers and mobile phones and the overall usage time of the three devices (all *p* < 0.01). People with obesity spent a short time using computers (2.38 vs. 2.07, *p* < 0.05) and mobile phones (3.00 vs. 2.60, *p* < 0.01). They also had a short overall time using the three devices (*p* < 0.001).

**Table 3:** Associations between BMI and obesity the time spent by the participants using the three electronic media devices.

	**TV program viewing time (daily hours), mean (SD)**	***P***	**Computer usage time (daily hours), mean (SD)**	***P***	**Mobile phone usage time (daily hours), mean (SD)**	***P***	**Overall usage time of the three devices (daily hours), mean (SD)**	***P***
**BMI (kg/m**^**2**^**)**		0.2		<0.001		<0.001		<0.001
<18.5	0.97 (1.03)		3.28 (3.10)		3.30 (2.14)		7.34 (4.10)	
18.5–23	1.16 (1.23)		2.43 (2.67)		3.12 (2.54)		6.37 (4.01)	
23–25	1.20 (1.22)		2.07 (2.45)		2.64 (2.31)		5.59 (5.60)	
≥25	1.14 (1.15)		2.07 (2.70)		2.60 (2.21)		5.40 (3.83)	
**Obese**		0.72		0.03		0.002		<0.001
Yes	1.14 (1.15)		2.07 (2.70)		2.60 (2.21)		5.40 (3.83)	
No	1.16 (1.22)		2.38 (2.66)		3.00 (2.47)		6.32 (3.96)	

### Multiple Linear Regression Results of BMI With the Three Electronic Media Devices

Table 4 shows the linear regression analysis results of BMI with the three electronic devices. We found no significant association between TV program viewing time and BMI (*p* > 0.05). By contrast, we observed significant inverse associations between BMI and computer and mobile phone usage in the simple regression model (both *p* < 0.05). The overall time of using the three devices also had a significant association with BMI [β = −0.08, 95% confidence interval (CI) = −0.109 to −0.05, *p* < 0.001]. Age, gender, work status, marital status, education level, family monthly income, drinking habits, and breakfast frequency had significant associations with BMI and the usage of the three electronic screen devices (*p* < 0.05). Hence, we introduced them into the multiple linear regression models as potential confounders. After adjusting for age and gender, the regression coefficient of computer usage was −0.093 (95% CI = −0.138 to −0.049, *p* < 0.001). This value had slightly increased after further adjusting for work status, marital status, education level, family monthly income, drinking habits, and breakfast frequency (β = −0.063, 95% CI = −0.112 to −0.015, *p* < 0.05). For the association between mobile phone usage and BMI, we found no significant association after adjusting for all confounders (*p* > 0.05). Regarding the total usage time of the three electronic devices usage, an hour increase predicting 0.04 kg/m^2^ decreases BMI (95% CI = −0.073 to −0.007, *p* < 0.05).

**Table 4:** Multiple linear regression results of BMI with the time usage of the three electronic media devices.

	**β**	**95% CI**		***P***
		**Lower bound**	**Upper bound**	
**Model 1**				
TV–BMI	0.06	−0.039	0.159	0.236
Computer–BMI	−0.121	−0.167	−0.076	<0.001
Mobile phone–BMI	−0.088	−0.138	−0.039	<0.001
Overall–BMI	−0.08	−0.109	−0.05	<0.001
**Model 2**				
TV–BMI	−0.008	−0.105	0.089	0.896
Computer–BMI	−0.093	−0.138	−0.049	<0.001
Mobile phone–BMI	−0.055	−0.103	−0.006	0.028
Overall–BMI	−0.056	−0.085	−0.026	<0.001
**Model 3**				
TV–BMI	−0.013	−0.121	0.094	0.805
Computer–BMI	−0.063	−0.112	−0.015	0.01
Mobile phone–BMI	−0.036	−0.088	0.017	0.181
Overall–BMI	−0.04	−0.071	−0.009	0.012
**Model 4**				
TV–BMI	−0.011	−0.12	0.097	0.837
Computer–BMI	−0.056	−0.105	−0.008	0.024
Mobile phone–BMI	−0.047	−0.1	0.005	0.077
Overall–BMI	−0.042	−0.074	−0.011	0.008

*Model 1: Simple model*.

*Model 2: Adjusted for age and gender*.

*Model 3: Adjusted for age, gender, work status, marital status, education level, and family monthly income*.

*Model 4: Adjusted for age, gender, work status, marital status, education level, family monthly income, drinking habits, and breakfast frequency*.

## Discussion

In our study, watching TV programs and using computers and mobile phones were all popular in Nanshan residents of Shenzhen. Mobile phones were the most frequently used electronic device in this population with the longest usage time. Each person reported an accumulated time of using the three devices beyond half of the daytime (>6 h). The age groups polarized the usage pattern. Young people spent less time watching TV programs but more time using computers and mobile phones. Unlike most previous studies, we found an inverse association between BMI and electronic device usage time, indicating that people who spent a long time using these three electronic devices were likely to have low BMI.

Our study sample comprised young people. Only 3% of the sample was elderly. Shenzhen is a rapidly developing city in South China, and it is one of the earliest open special economic zones, with a high degree of outward orientation in terms of economy. Many big information technology companies, such as Huawei, Tencent, and ZTE, are in Shenzhen. It caters to many immigrants, and thus well-educated young people go to this city from other relatively underdeveloped provinces to seek for a job, with a dream of achieving future goals. It is a typical characteristic of Shenzhen residents, which is different from other cities in China. It reflects a common phenomenon in a fast-growing city of new immigrants. Following this logic, we were not surprised to find that young population dominated our sample. The young adults usually have an extensive social network with more social support (20, 21). They may spend their free time with friends or colleagues at social gatherings than watching TV programs (7, 22). Also, these young adults might use electronic devices more for work and exercise than for sedentary entertainment such as playing video games. These could explain why we found an inverse association between overall device usage time and BMI.

The average hours of TV program viewing in our study were shorter than those in other areas/regions of China, such as Hong Kong (10) and Nanjing (23), and countries such as the United States (24), Canada (25), Japan (26), and Belgium (27). Shenzhen is highly open, with a developed trade industry. E-government and e-commerce had become the characteristics of this city wherein the Internet is indispensable in the residents' life. Thus, mobile phone and computer usage were ubiquitous in our study sample. We found no significant association between TV viewing time and BMI and obesity, which was inconsistent with many previous studies (7, 28, 29). Only a few studies found no significant association between TV viewing time and BMI (13, 30, 31). The different measurements for TV viewing and the variety of the population might be the reasons. Our study sample comprised young people, and thus, TV program viewing was not a major entertainment during their leisure time. This reason could explain why we did not find any significant association. Another possible explanation is that self-reported TV viewing time could be subject to social desirability and social approval bias, given that some individuals might have chosen to underreport their sedentary behaviors (32).

After adjusting the covariates, we also found no association between mobile phone usage time and BMI. People used mobile phones when waiting for public transportation or traveling, laying on the bed, or during sitting. Given that people are not always sedentary when using mobile phones, we observed no significant association between mobile phone usage and BMI in our study and some previous studies (33, 34).

Surprisingly, we found that long computer usage time predicts low BMI. Using computers is sedentary behavior, but we found an inverse association, which was contrary to many previous studies (16, 35, 36). Given that young people dominated our study sample, the following characteristics of such a population might affect our results: Young people may use electronic devices more for work and exercise than for passive entertainment, such as playing video games. Furthermore, they could have done various compensatory exercises/movements. Internet addiction (37) could be another reason that leads to less consumption of food during computer usage (38). As for those who use computers for a long time at work, psychosocial stress from job strain may result in weight loss from diminished appetite (39–41).

These reasons might explain the identified inverse associations in our study. We also observed a similar inverse association when we calculated for the sum of the usage time of the three electronic devices, indicating that some mediators unique in our study population could lead to such inverse associations. Accordingly, scholars should conduct further investigations. Our study results revealed a different finding toward most previous studies, which could capture attention in this research area and inspire researchers for additional insights. We suggest that future works should consider and use more detailed usage pattern and behavioral factors unique in young people.

This study had several limitations. First, we did not obtain detailed data on measuring the use of the three electronic devices, such as the purpose for the use (e.g., for Internet access or playing games), preferred time of using such technologies, and information the people gain from such an activity. Future studies could make more insights into these aspects to assess the usage pattern of the three electronic devices comprehensively. Secondly, we had access only to limited dietary intake data and had no information on the length of sedentary bouts or sleep duration, as well as consumption of fruits and vegetables. All these factors may contribute to changes in BMI and the development of obesity, in addition to electronic device usage. Further studies may explore the relationships by comprehensively assessing these lifestyle factors. Third, we only conducted a cross-sectional study, which could not determine the causal relationship. Fourth, our data collection relied on self-administered measures, which might lead to self-report bias to some extent. Still, we suggest that it would not overturn the associations we found. Our sample was large enough, making the overestimation or underestimation in self-reporting as random. To the best of our knowledge, we are among the first population-based studies to examine the pattern of the time usage of the three common screen-based electronic devices in a Chinese adult population comprehensively and investigate their associations with BMI and obesity. In this study, BMI had inverse associations with computer usage time and the overall usage time of the three electronic devices. This finding suggests that using screen-based devices at a specific level would not be a risk factor for developing such health problems in this young population. Although we did not find a negative effect of electronic device usage on BMI/obesity, we still suggest that people should appropriately and moderately use screen-based electronic devices. We also strongly encourage future studies to conduct a longitudinal observation of the detailed pattern of electronic device usage and its associations with various health conditions.

## Data Availability Statement

The raw data supporting the conclusions of this article will be made available by the authors, without undue reservation.

## Ethics Statement

The Huazhong University of Science and Technology Union Shenzhen Hospital and the Human Research Committee of Hong Kong Polytechnic University approved the study (HSEARS20180521004). All participants signed the informed consents.

## Author Contributions

YJX and BM: conceived and designed the study. HY, ZH, and PL: collected the data. YJX, YQ, and LC: analyzed the data. YJX, YQ, SLW, and LC: wrote the paper. All authors: contributed to the article and approved the submitted version.

## Conflict of Interest

The authors declare that the research was conducted in the absence of any commercial or financial relationships that could be construed as a potential conflict of interest.
